# RFFE – Random Forest Fuzzy Entropy for the classification of Diabetes Mellitus

**DOI:** 10.3934/publichealth.2023030

**Published:** 2023-05-23

**Authors:** A. Usha Ruby, J George Chellin Chandran, TJ Swasthika Jain, BN Chaithanya, Renuka Patil

**Affiliations:** 1 School of Computing Science and Engineering Department, VIT Bhopal University, Bhopal-Indore Highway, Kothrikalan, Sehore, Madhya Pradesh–466114, India; 2 Department of Computer Science and Engineering, GITAM School of Technology, Nagadenehalli, Doddaballapura, Karnataka–561203, India

**Keywords:** diabetes diseases, Fuzzy Entropy, machine learning, Synthetic Gradient Descent Technique

## Abstract

Diabetes is a category of metabolic disease commonly known as a chronic illness. It causes the body to generate less insulin and raises blood sugar levels, leading to various issues and disrupting the functioning of organs, including the retinal, kidney and nerves. To prevent this, people with chronic illnesses require lifetime access to treatment. As a result, early diabetes detection is essential and might save many lives. Diagnosis of people at high risk of developing diabetes is utilized for preventing the disease in various aspects. This article presents a chronic illness prediction prototype based on a person's risk feature data to provide an early prediction for diabetes with Fuzzy Entropy random vectors that regulate the development of each tree in the Random Forest. The proposed prototype consists of data imputation, data sampling, feature selection, and various techniques to predict the disease, such as Fuzzy Entropy, Synthetic Minority Oversampling Technique (SMOTE), Convolutional Neural Network (CNN) with Stochastic Gradient Descent with Momentum (SGDM), Support Vector Machines (SVM), Classification and Regression Tree (CART), K-Nearest Neighbor (KNN), and Naïve Bayes (NB). This study uses the existing Pima Indian Diabetes (PID) dataset for diabetic disease prediction. The predictions' true/false positive/negative rate is investigated using the confusion matrix and the receiver operating characteristic area under the curve (ROCAUC). Findings on a PID dataset are compared with machine learning algorithms revealing that the proposed Random Forest Fuzzy Entropy (RFFE) is a valuable approach for diabetes prediction, with an accuracy of 98 percent.

## 1. Introduction

The world faces a significant medical and economic impact because of the rapid rise of diabetes during the last few decades. The public health problem of diabetes is significant. Many people with diabetes globally, from diverse socioeconomic and racial groups, are affected by the disease. 2008 [1] Ambady et al. Diabetes is a significant public health issue. Worldwide, diabetes affects many people from different socioeconomic and racial backgrounds. Diabetes is a complex metabolic disease that can devastate a person's life and damage many bodily systems and organs. Cardiovascular illness can strike diabetics up to four times more frequently than non-diabetics, and lumbar spine surgery can happen up to 40 times more frequently in diabetics. In adults, diabetes is one of the leading causes of visual loss, glaucoma, and renal disease.

Due to the early disability, morbidity, and death it causes, diabetes is one of the most expensive diseases to treat. This places additional strain on people and families, society, and the country's healthcare system Khuwaja et al., 2010 [2]. The best way to avoid various complications is to detect the disease earlier. Many studies have been conducted on early diabetes predictions, including diagnosis, categorization, and medication Kumari S et al., 2021 [3]. Researchers have done experimental studies to diagnose diabetes illness by employing various Machine Learning (ML) classification algorithms such as J48, SVM, Naive Bayes, Decision Tree, Decision Table, and others. To the studies, Data Mining and Machine Learning techniques can manage a vast quantity of data, aggregate data from several sources, and integrate background information Sisodia et al., 2018 [4]. Data mining and machine learning algorithms have proven to be precise tools in the computer science field and are widely used in several fields. Medical science is one of these fields. Especially for extracting hyperparameters, selecting the features, and mining critical and valuable clinical data.

In the field of medical diagnostics, researchers have developed a variety of strategies in recent years. Diabetes Mellitus (DM) is one of the hundreds of diseases that is rapidly expanding worldwide. DM has appeared as a disease requiring immediate care due to its fast spread worldwide (Reddy et al., 2019 [5]). Researchers are still developing a generic model that can anticipate the kind of ailment a diabetes patient will experience. Different ML and Neural Network-based diabetes forecasting methodologies and strategies have been reported in the literature based on distinct models. These approaches extract, evaluate, and interpret the available diabetic data to make diagnoses.

**Figure 1.**
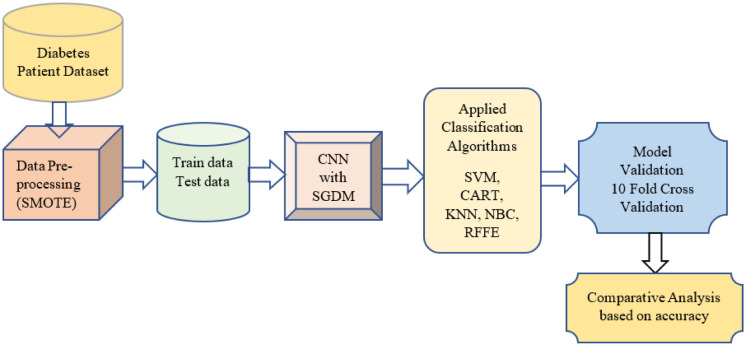
Proposed RFFE model.

Figure 1 depicts a proposed RFFE model for such strategies. The most current studies on DM categorization are reviewed in this paper. Overall, this research focuses on using Deep learning approaches for DM classification and their influence on classification outcomes. Based on the underlying model, the frequently used ML and Convolutional Neural Network-based solutions for diabetes diagnosis and prediction are categorized with Fuzzy Entropy. Comparison is made using 10-Fold Cross Validation performance for the used algorithms.

## 2. Related work

Various works of literature have promoted disease identification and estimation research, extending the improvement and implementation analysis of ML and Neural network algorithms for diabetes disease finding, forecasting and categorizing. Taiyu Zhu et al., 2020 [6] gave a detailed overview of deep learning applications in diabetes. They did a thorough literature search and discovered three key areas where this technique is used: diabetes diagnosis, glucose management and diabetes-related problems. The search yielded 40 original research publications that summarized critical information regarding the used learning models, development process, primary outcomes and performance evaluation baseline methodologies. On Imbalanced data with Missing values, Qian Wang et al., 2019 [7] suggested an excellent Prediction method for Diabetes Mellitus categorization. The missing values are first compensated using the Nave Bayes (NB) approach for data normalization. Then, an adaptive synthetic sampling approach is used to minimize the impact of class imbalance on prediction performance. Finally, predictions are generated using a random forest (RF) classifier and assessed using a complete set of evaluation indicators. In their study, Nahla H. Barakat et al., 2010 [8] recommended using support vector machines (SVMs) to diagnose diabetes. The author employed an explicit explanation module to transform an SVM's “black box” model into an understandable representation of the diagnostic (classification) conclusion. Results on a real-world diabetes dataset reveal that intelligible SVMs are a viable tool for diabetes prediction, with an understandable ruleset and prediction accuracy of 94 percent, sensitivity of 93 percent and a specificity of 94 percent.

Yu Wang et al., 2016 [9] proposed a shared decision-making context for type 2 diabetes mellitus (T2DM) patients that includes not only extracting information from standards that uses class-imbalanced electronic clinical records and aims to provide a recommended medication to support doctors and patients in having a shared decision-making conversation. The recommendation model performed exceptionally well as a complete multilabel classifier, with Hamming Loss values of 0.0941, Accuracyexam ratings of 0.7611, Recallexam scores of 0.9664 and Fexam scores of 0.8269. A multi-view convolutional neural network classification model based on inceptionV1 was proposed by Dong Wen et al., 2020 [10] to improve the performance of convolutional neural networks in EEG multispectral picture categorization. The convolution layers and stochastic gradient descent in the convolution model are primarily enhanced and optimized. According to the findings, the proposed model offered superior stability and accuracy to standard classification models. Evanthia E. Tripoliti et al., 2011 [11] introduced a model for producing an accurate and varied ensemble, assuring the two crucial features an ensemble classifier should have. This approach is based on an online fitting procedure and it is tested on eight biomedical datasets and five random forests algorithm versions (40 cases). In 90% of the test scenarios, the approach adequately determined the number of trees. A. Usha Ruby et al., 2022 [12] suggested an efficient parameter signifier method to classify plant leaf disease using various machine learning algorithms.

Chun Ouyang et al., 2021 [13] created a layered multi-task fusion convolution neural network for feature detection, which was trained in 30 minutes using our server. In 9 of 12 situations with analytically adjusted hyperparameters, the proposed layer outperformed the single-task convolution neural network in classification accuracy. The greatest accuracy was 90.6 percent with a threshold of 6000, comparable to the accuracy of diabetes classification methods. Marco Recenti et al., 2020 [14] investigated how previous and current lifestyle impacts the occurrence of comorbidities such as hypertension, diabetes and heart disease. It was categorized into three levels: 1) behavioral factors (smoker and self-reported insufficient physical activity), 2) comorbidity hypertension or diabetes and 3) cardio pathology. Differences were explored on every level between the categories, and tree-structured machine learning classifications were used to categorize participants with hypertension or diabetes. The scores for identifying hypertension or diabetes based on daily life characteristics were highly accurate, with ROCAUC 97.8% and 99%, respectively). A CNN model to predict cardiac vascular events was designed by Enrico Longato et al., 2021 [15]. It signifies the 4P- significant adverse cardiovascular events such as the first incidence of fatality, cardiac arrest, coronary artery disease, or hemorrhage using a year of pharmaceutical and hospitalized records and essential clinical records with a flexible simulation period of 1 to 5 years. At all prediction horizons, the model performs satisfactorily in predicting 4P- significant adverse cardiovascular events. S. Lekha et al., 2017 [16] investigate using a one-dimensional convolutional neural network approach incorporating feature extraction and classification techniques. The strategy suggested in this study is found to greatly lessen significantly the restrictions associated with utilising these strategies separately, further increasing the classifier's performance. This work proposes using a modified 1-D CNN to breathe data received from an array of gas sensors. The system's performance and experiments are carried out and assessed.

Shu-Chen Cheng et al., 2003 [17] presented a unique diagnostic approach for developing quantitative diabetes indices. Because the author observed that the fractal dimension of an acute diabetic-affected person's retinal vascular dissemination is higher than that of an unaffected person, the fractal component of the vascular dissemination was calculated. Four distinct ways to categorize diagnosis results are examined to improve accuracy. To assess and filter the most important diabetics chance factor for Type 2 Diabetes mellitus prediction, Asif Hassan Syed et al., 2020 [18] applied data imputation and augmentation. The cross-sectional data was balanced using SMOTE, a class-balancer. The hyper-parameters of the best-performing classifier were fine-tuned using 10-fold cross-validation to increase the F1 Score. The tweaked two-class Decision Forest model performed better with an average F1 score of 84.53 percent to 2.68 percent. Mohammad Z. Atwany et al., 2022 [19] proposed retinal fundus picture categorization and detection after reviewing and analyzing deep learning approaches in various transformer settings. For example, the categories of Diabetes Retinopathy are assessed and summarized as referable, non-referable and proliferative. Furthermore, the research analyses the existing Diabetic Retinopathy retinal fundus datasets for various tasks such as identification, categorization and prediction. Multiple investigations reveal an average accuracy of around 91 percent and overall promising categorization performance.

Konstanze Kolle et al., 2019 [20] devised an automatic meal detection system that might free up the user and enhance glucose control. In this investigation, features in postprandial continuous glucose monitoring data are used to detect meals. In horizons of the predicted glucose rate of appearance and continuous glucose monitoring data, binary classifiers are built to detect the postprandial pattern. Cross-validation was used to validate the categorization. Linear discriminant analysis outperformed threshold-based approaches regarding meal sensitivity and false alarm rate. Zarkogianni et al., 2015 [21] discussed that the change toward preventing, predicting, customizing and participating in diabetic treatment is enabled by combining data from the Internet of things-based systems and digital clinical records with big data analytics. The possibility of precepting and predictive modeling techniques for enhancing diabetic treatment and the limitations accompanying them are discussed. The suggested study by S. Gayathri et al., 2020 [22] focuses on binary and multiclass categorizing diabetic retinal disease. The system is tested using picture characteristics taken from three sets of databases. The various metrics of each classification algorithm are compared. According to the assessment findings, Random Forest surpasses several classification models with a median precision of 99.7 percent for binary classifiers and 99.82 percent for multiclass classifiers when using the suggested feature extraction approach.

Mohammad Tariqul Islam et al., 2021 [23] developed a unique computational intelligence architecture to predict diabetes based on retinal images. The author builds a multi-stage, fully CNN-based model DiaNet, which can achieve an accuracy level of over 84 percent. Furthermore, the findings suggest that retinal pictures may include prognostic indicators for diabetes and other comorbidities. For widespread diabetes diagnosis, Hongxu Yin et al., 2019 [24] advocated DiabDeep, a system that blends efficient neural networks (known as DiabNNs) with wearable medical sensors. DiabDeep works directly on wearable medical sensing data, bypassing the feature extraction stage. It allows for (a) precise inference on the servers and (b) efficient inference on devices like mobile phones. A rigorous examination of data acquired from 52 individuals is used to illustrate the performance of DiabDeep. The author obtained the result of 96.3% accurate identification of diabetic versus healthy people on the servers and 95.7% accuracy in discriminating between type1 diabetic, type2 diabetic and normal people. Julian Theis et al., 2021 [25] built a process with data mining and deep learning architecture that incorporates the medical history of diabetic patients to augment conventional severity grading methodologies. First, past medical health records are transformed into events logs suited to mine the data. The events logs are then utilized to create a processes model which defines patients' previous clinical records. It is used to modify Decays Replayed data mining to blend clinical and demographics data along with existing simplicity scores to forecast hospital death in diabetic intensive care unit patients.

Kamrul Hasan et al., 2020 [26] In their article, a subjective ensemble of several ML models are proposed to enhance diabetes prediction, with the weights calculated from the ML model's corresponding areas in the receiver operator characteristics curve. The performance metric is determined from the areas in the receiver operator characteristics curve, which is then maximized during hyperparameter tuning using the grid search approach. The Pima Indian Diabetes Dataset was used to conduct all the research in this literature under identical experimental circumstances. The suggested ensembled classifier shows the best performance with various metrics of 78.9% in sensitivity, 93.4% in specificity, 9.2% in false omission rate, 66.23% in diagnostic odds ratio and 95% in ROCAUC respectively, constructed on all the comprehensive trials. In cloud computing, P. G. Shynu et al., 2021 [27] proposed an efficient, decentralized-based, secure clinical care service for illness estimation. Diabetics and cardiac illnesses are considered when making predictions. The patient's clinical data is initially acquired through an intermediate node and kept in a decentralized system. Initially, the innovative cluster technique based on rules was used to cluster patients' clinical information. Finally, a feature collection based on a neural fuzzy reasoning method is used to predict diabetes and cardiovascular illnesses (FS-ANFIS). Compared to existing neural network techniques, the suggested approach has a prediction accuracy of over 81 percent.

Nada et al., 2022 [28] developed an extensive data analytic suite for type 2 diabetic infection management that helps doctors and scholars to find links between distinct patients' biological indicators and type 2 diabetic-associated problems. The big data analytical package includes visuals and predictions with features like the multiple-tier categorization of type 2 diabetes patients' profiles that link them to precise illnesses, type 2 diabetes associated with complicated risk estimation, and patient response forecast to a specific treatment method. Based on three categories of characteristics retrieved from the tongue image dataset, Bob Zhang et al., 2013 [29] suggested a noninvasive approach to diagnose diabetes radiotherapy and no proliferative diabetic retinopathy, the earliest stage of diabetes radiotherapy. The geometry features comprise 13 characteristics retrieved from tongue pictures based on measures, distances, areas and ratios. Utilizing a combination of the 34 characteristics, the proposed technique can distinguish between Healthy/diabetes radiotherapy tongues and no proliferative diabetic retinopathy tongues with median accuracies of 80.52 percent and 80.33 percent, respectively, using features from each of the three categories. Bum Ju Lee et al., 2013 [30] intended to forecast the fast blood plasma insulin status applied in analyzing type 2 diabetics among adults in Korea. 4870 sample data (2955 female and 1915 male) contributed to this analysis. Established on thirty-seven anthropometrical rates, the author compared the prediction of fasting plasma glucose levels using specific versus blended rates using two machine classification algorithms. The principles of the areas in the receivers operate characteristic curves for the predictions by logistical regressions, and Naïves Baye's classifiers based on the mixture of procedures were 74.1% and 73.9% in female data, respectively, and were 68.7% and 68.6% in male data, correspondingly.

Farrukh Aslam Khan et al., 2021 [31] presented a comprehensive review of diabetes diagnosis and prediction using data mining. This paper aims to explore and investigate the data mining-based diagnosis and prediction solutions in glycaemic control for diabetes. Pratya Nuankaew et al., 2021 [32] devised a Median Weight Objectives Distant for binary classifications problem. Datasets from open source, Pima Indians Diabetes (Dataset 1) and Mendeley Data for Diabetes (Dataset 2), each having three hundred and ninety-two entries, were investigated to validate the suggested technique. According to the comparative findings, the suggested approach delivered 93.22 percent and 98.95 percent accuracy for Dataset1 and Dataset2 more significantly than existing machine learning-based methods. Anas Bilal et al., 2021 [33] suggested a unique and multimodal approach for detecting and classifying prior diabetic retinopathy. Pre-processing feature extraction and classification methods are followed in the proposed study. The pre-processing stage improves anomaly detection and segmentation; the extraction step only extracts essential characteristics, and the classification step employs a variety of classifiers. Multiple severities of illness grading databases were used to complete this research, which resulted in 98.06 percent accuracy, 83.67 percent sensitivity and 100 percent specificity.

Amparo Güemes et al., 2019 [34] presented a method for predicting whether nocturnal blood glucose concentrations will stay within or beyond the desired range, allowing the user to take the necessary preventive action. On a publicly available clinical dataset, various commonly established machine learning methods for binary classification were studied and compared (OhioT1DM dataset). According to this study, it is feasible to predict the quality of night-time glycaemic control with an acceptable accuracy of 70% by utilizing routinely collected data in type 1 diabetic treatment. Bob Zhang et al., 2013 [35] suggested a new noninvasive technique based on face block color characteristics and a sparse-representation-classifier to identify diabetes mellitus. Initially, a picture comprising four facial blocks strategically arranged around the face is captured using noninvasive capture equipment with image correction. The sparse-representation-classifier procedure for SRC uses two sub-dictionaries: a healthy facial color features sub-dictionary and a diabetic mellitus facial color features sub-dictionary. The findings of an experiment with 142 normal and 284 diabetic mellitus samples are displayed. The sparse-representation-classifier can discriminate between normal and diabetes mellitus classes with a median accuracy of 97.54 percent using a mixture of face blocks.

Tuan Minh Le et al., 2020 [36] proposed an ML approach for predicting diabetes patients' development early. It's a new wrapper-based features selection method that employs the Grey-Wolf-Optimize and Adaption Particles Swam method to optimize the Multi-layered Perceptron and decrease the needed input feature attributes. The suggested method's computational findings demonstrate that fewer characteristics are required, and greater prediction accuracy (96 percent for Grey-Wolf-Optimize-Multi-layered Perceptron and 97 percent for Adaption Particles Swam-Optimization-Multi-layered Perceptron) can be reached. Maryamsadat Shokrekhodaei et al., 2021 [37] The custom-built optical sensor is investigated in this work, utilizing approximately 18 distinct wavelength ranges between 400 and 900 nm. The results demonstrate approximately a substantial association value (0.97) for four wavelengths between glucose levels and transmission intensities (480, 640, 860 and 940 nm). For glucose predictions, various machine classification methods are studied. When regression techniques are utilized, 9% of glucose forecasts are off by a factor of two (normal, hypoglycaemic, or hyperglycaemic). Feature Classifications-based model surpasses the regression model, and the support vector machines, with an F1-score of 99 percent.

## 3. Experimental methodology

### 3.1. Dataset

**Figure 2.**
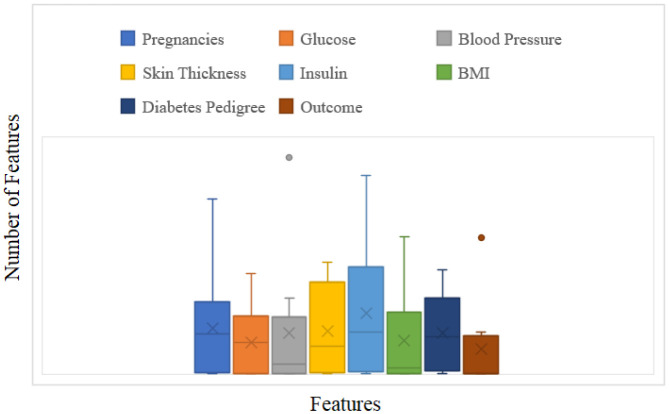
Distribution of sample data in PID dataset.

The PID Database of the National Institute of Diabetes and Digestive and Kidney Diseases was used in this work. Vincent Sigillito provided this diabetes database, which comprises 768 medical diagnostic records from a community around Phoenix, Arizona, in the United States. The samples include instances with eight attribute values and one of two potential outcomes: whether the patient is diagnosed with diabetes (indicated by output one) (indicated by zero) or not. Much research has made use of this freely available PID dataset. It has 768 examples, each with 8 characteristics and a binary label (0 or 1). For learning and testing data, stratified 10-fold cross-validation is employed. This implies that the learning process is repeated ten times after being divided into equal portions of the training data. Each time, a different dataset component is selected for testing while using the remaining nine components for learning. A stratified 10-fold cross validation is used since it is now the most effective and up-to-date approach for validating data. The dataset was split into training data for developing the classification model and test data for assessing the model's implementation. The training data to test data ratio is 8:2. Table 1 provides an overview of the patient data with and without diabetes disease for ten patients with the features like Pregnancies, Glucose, Blood Pressure, Skin Thickness, Insulin, BMI (Body Mass Index), Diabetes pedigree function, Age and Outcome. We selected the following independent variables-Pregnancies, Glucose, Blood Pressure, Skin Thickness, Insulin, BMI (Body Mass Index), Diabetes pedigree function; and Outcome is the dependent variable. The variables were selected based on their established associations with the development and progression of diabetes, as well as their availability in the Pima Indians Diabetes dataset. Pregnancies, Glucose, Blood Pressure, Skin Thickness, Insulin, BMI (Body Mass Index) and Diabetes pedigree function have all been shown in previous research to be important risk factors for the development of diabetes. For example, studies have shown that high glucose levels, high blood pressure and obesity are associated with an increased risk of diabetes [47]. In addition, the number of pregnancies and the diabetes pedigree function have been shown to be important risk factors for the development of diabetes in certain populations [48]. The distribution of the sample data in the PID dataset for the features is depicted in Figure 2. The dataset used is taken from Kaggle, named as the “[Global Dataset] Pima Indians Diabetes” [49].

**Table 1.** A sample of the PID dataset.

	Pregnancies	Glucose	Blood Pressure	Skin Thickness	Insulin	BMI	Diabetes Pedigree	Age	Outcome
Patient 1	6	148	72	35	0	33.6	0.627	50	1
Patient 2	1	85	66	29	0	26.6	0.351	31	0
Patient 3	8	183	64	0	0	23.3	0.672	32	1
Patient 4	1	89	66	23	94	28.1	0.167	21	0
Patient 5	0	137	40	35	168	43.1	2.288	33	1
Patient 6	5	116	74	0	0	25.6	0.201	30	0
Patient 7	3	78	50	32	88	31	0.248	26	1
Patient 8	10	115	0	0	0	35.3	0.134	29	0
Patient 9	2	197	70	45	543	30.5	0.158	53	1
Patient 10	8	125	96	0	0	0	0.232	54	1

We used the Pima Indians Diabetes dataset from Kaggle, which contains **768 observations** of diabetic and non-diabetic patients. While we understand that the sample size may not be as large as some other datasets in the field, we believe that it is appropriate for our research question. To support this, we performed a **power analysis** to determine the minimum sample size required to detect a statistically significant difference between the diabetic and non-diabetic groups. Based on this analysis, we found that the sample size of ***768 was sufficient*** to detect a meaningful difference with a ***power of 0.80 and a significance level of 0.05*.** The formula used in Eq 1 for power analysis is as follows:

$$\begin{array}{c} n=2{\left(Z_{\text{α}/2}+Z_{\text{β}}\right)}^{2}\frac{s^{2}}{d^{2}} \end{array}$$ (1)

- *n* is the required sample size.
- *Z_α_*_/2_ is the critical value of the standard normal distribution at the alpha level of significance (0.05/2 = 0.025 for a two-tailed test).
- *Z_β_* is the critical value of the standard normal distribution at the desired power level (0.80 in our study).
- *s* is the standard deviation of the outcome variable (diabetes status) in the population.
- *d* is the difference in the mean outcome variable between the two groups (diabetic and non-diabetic).

In addition, we acknowledge the potential limitations of our study, including the sample size. We have included a section on limitations in our paper, where we discuss the potential impact of the sample size on our results and conclusions. We believe that by acknowledging these limitations and being transparent about the potential impact of the sample size, we can provide a more accurate and informative account of our research.

### 3.2. Data pre-processing and sampling

The proposed Random Forest Fuzzy Entropy approach, based on sampling clustering of feature sets, considers the correlation and non-correlation of selected features in the dataset. We have collected the selected features for the minority of classes by using clustering and oversampling methods. Oversampling was utilized to solve the problem of a highly skewed class distribution, making it difficult for learning algorithms to develop good models as discussed by Chawla et al., 2002 [38].

Furthermore, reducing the number of negative instances in the training set increases the sensitivity of the learned model to false-positive classifications. Therefore, utilizing a sampling method wherein the minority class is over-samples by constructing “synthesized” instances instead of over-sampled data using replacement. Mukherjee et al., 2021 [39] SMOTE (Synthetic Minority Oversampling Technique), is used for a pre-set number of neighbours, which were estimated for each underrepresented instance in the dataset. Then specific random minority class examples were chosen for synthesized data point production.

**Figure 3.**
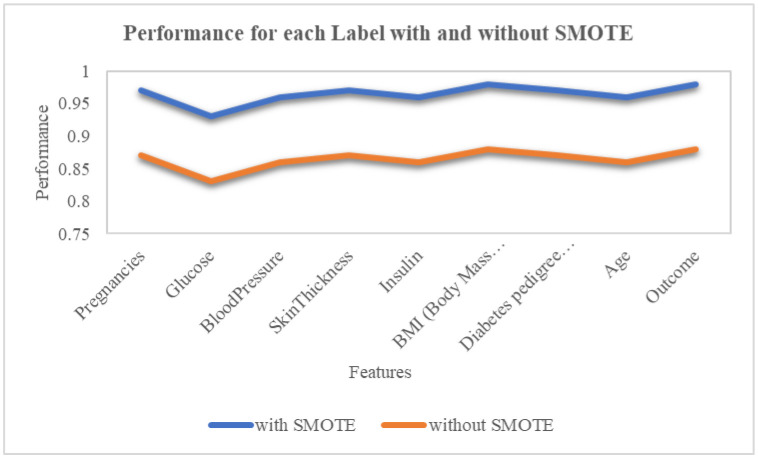
Performance with and without SMOTE.

**Figure 4.**
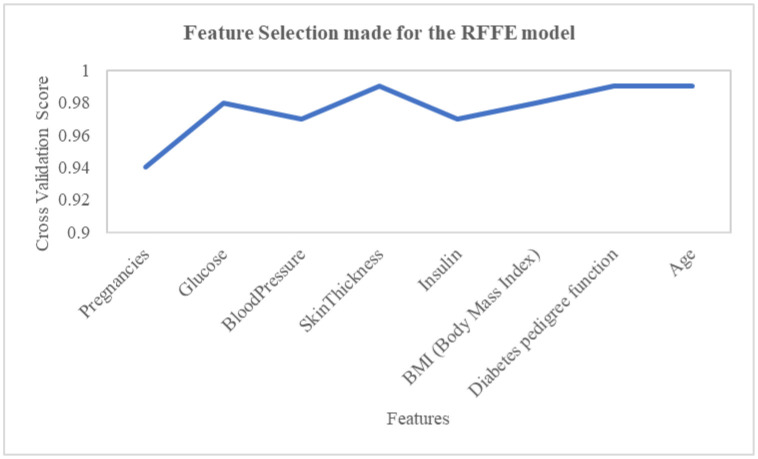
Feature selection.

Thus, fabricated observation was made all along the line-up, separating the chosen minority occurrence from its nearest neighbours. SMOTE treated the nominal attributes differently from continuous attributes and kept the original labels of definite features in the resampled data, which was used to detect insignificant features. Applying SMOTE in this work makes the machine learning algorithms anticipate the underrepresented events with significant accuracy. The performance curve with and without SMOTE is illustrated in Figure 3. The feature selection with cross-validation score for the proposed model which is used to increase the accuracy is depicted in Figure 4.

### 3.3. Convolutional neural network

CNN has been widely used in diabetes illness categorization, yielding several notable study findings for example Chaithanya BN et al., 2021 [40], Aslan et al., 2021 [41]. Following the oversampling of data, the new data is modelled using CNN. Forward propagation, reverse propagation of errors, computing the prediction error and updating the parameter matrix are the four basic processes in the training/learning process. Furthermore, SGDM is the optimization approach used for parameter training and updating for the proposed model. In Eq 2, the SGDM optimization algorithm's parameter update formula is presented.

$$\begin{array}{c} \theta_{i+1}=\theta_{i}-\alpha \nabla E\left(\theta_{i}\right)+\gamma \left(\theta_{i}-\theta_{i-1}\right) \end{array}$$ (2)

Here, *θ* refers updated feature, *α* refers to the learning rate and *γ* denotes the momentum value. Table 2 lists the parameters of this optimization process, including Momentums, Starting Learning Rate, Epoch value and Batch Size. The backpropagation method updates the network's weights in each iteration. The output from the fully connected layer is further used for machine learning classifiers. In RFFE model, the first Conv2D layer had 16 filters, followed by two more Conv2D layers with 32 and 64 filters respectively.

**Table 2.** Training Parameters of implemented CNN models.

Maximum Epoch	Minimum Batch Size	Learning Rate (*α*)	Momentum (*γ*)	Kernal Size
100	30	0.009	0.94	1 x 1

### 3.4. Support vector machines

Schuldt et al., 2004 [42] stated that Support Vector Machines (SVMs) are high- performance, more significant margin classifiers increasingly evolving in machine learning technology. The supervised machine learning model, or SVM, is a subset of the supervised machine learning model. It's best suited to a small data collection with fewer outliers. Our goal is to find a hyperplane that will allow data points to be separated. This hyperplane (HN) will split space into domains, each containing a distinct form of data. Consider the possibility of categorizing a training data (a_1_, b_1_), (a_2_, b_2_), ... (a_m_, b_m_) into two classes, with a_i_ Є HN as the features vector and b_i_ Є {1, + 1) as the class labels of m. The optimum hyperplane is the one that maximizes the margins, if a hyperplane w.a + d = 0 in any space HN may separate the classes with no preceding knowledge of the data distribution. Its best w and d values may be found by explaining a limited minimum problem with Lagrange multipliers α_i_ (i = 1, ... m) with α_i_ and d calculated using a Support Vector Classifier (SVC) learning approach by using the Eq 3. Therefore, the dataset's classification accuracy with the SVM classifier is 83 percent.

$$\begin{array}{c} f\left(x\right)=sgn\left(\overset{m}{\underset{i=1}{\sum }}\alpha_{i}b_{i}K\left(a_{i},b\right)+d\right) \end{array}$$ (3)

### 3.5. Classification and regression tree

Breiman et al., 1984 [43] introduced a classification and regression tree (CART) that builds binary trees. CART accepts data with numeric or categorical values and manages missing attribute values. Regression trees are generated via cost-complexity pruning. The CART approach is resistant to outliers. For attribute selection, we employed CART with entropy as the impurity measure. The property with the most significant impurity reduction separates the node's contents. The following Eqs 4 and 5 are used to discover the optimal features for the tree by using entropies and discriminative powers where *y_k_*, k = 1, 2, ... m are the features of the dataset. The accuracy derived from this classification algorithm for the PID dataset is 75 percent.

$$\begin{array}{c} Entropy:E\left(Y\right)=\overset{m}{\underset{k\;=1}{\sum }}P\left(y_{k}\right)*\log P\left(y_{k}\right) \end{array}$$ (4)

$$\begin{array}{c} Discriminative\;power=entropy\left(parent\right)-\left(weight\;average\right)*entropy \end{array}$$ (5)

### 3.6. K-Nearest Neighbor

KNN approach is used to classify diabetes disease. KNN is frequently used in the Data Mining field to categorize items based on the distance between the item (Query point) and all other objects in the Training Data discussed in this paper by Altman et al., 1992 [44]. An object's K neighbors are used to categorize it. K is determined to be a positive integer before the method begins. Euclidean distance is widely employed for estimating the distance between two objects. The Euclidean distance may be calculated using the following Eq 6 where *a_m_ and b_m_* are the query points.

$$\begin{array}{c} D_{Euclidean}\left(a,b\right)=\sqrt{\overset{n}{\underset{m\;=1}{\sum }}{\left(a_{m}-b_{m}\right)}^{2}} \end{array}$$ (6)

The following is a description of the KNN method:

- Calculate the K value (number of nearest neighbors).
- Determine how far the categorized item (Query Point) is from all other objects.
- Arrange the spaces in ascendingly and find the K Query Point's closest neighbors.
- Collect all courses from the nearest K neighbors.
- Determining the class for the Query Point based on most of the closest neighbor's class.

### 3.7. Naïve Bayes

Yager RR et al., 2006 [45] stated that the NB classifier is a probabilistic classification method. The classifier predicts that an unclassified feature y = (y_1_, ... y_n_) belongs to the category *C_i_* with the highest probability conditioned on y. Specifically, if and only if, this classifies features y into category *C_i_* as expressed in Eq 7. We may describe Bayes' theorem as depicted in Eq 8. The requisite probabilities can be determined using training samples. NB algorithm is used to create the predictions on a PID dataset, resulting in an accuracy percentage of 65.

$$\begin{array}{c} P\left(C_{i}\text{|}Y\right)>P\left(C_{j}\text{|}Y\right)\;for\;all\;j\neq i \end{array}$$ (7)

$$\begin{array}{c} P\left(C_{j}\text{|}Y\right)=\frac{P\left(X\text{|}C_{j}\right)P\left(C_{j}\right)}{P\left(Y\right)} \end{array}$$ (8)

### 3.8. Random Forest Fuzzy Entropy

Breiman L 2001 [46] discussed that Random Forest is a set of trees predictor in which every tree is defined by the value of a randomized vector selected individually and consistently throughout the forest tree. When the number of trees in a random forest grows longer, the generalization error ness converges to a maximum employed with fuzzy entropy. Developing an ensemble of trees with fuzzy entropy and allowing them to vote on the most popular class has significantly increased classification accuracy. Fuzzy entropy random vectors that regulate the development of each tree in the ensemble are constructed to grow the ensembles. In all these techniques, a fuzzy entropy random vector *θ_i_* is created for the ith tree, unbiased of the previous fuzzy entropy random vectors *θ*_1_ ... *θ_i_*. But with the same distribution; then a tree is formed using the training data and i, resultant in a classifier-C (y, *θ_i_*), where y is an input vector.

In bagging, for example, the fuzzy entropy randomized vector *θ* is created as the amount in M boxes because of M dart dropped in a randomized manner into the boxes, where M is the number of samples in the training data. A randomized split-up selection comprises many unbiased randomized integers ranging from 1 to *i*. Its dimensions and character are determined by how it is used in tree construction. In turn, it votes for the most popular class after producing several trees. These processes are known as random forest fuzzy entropy. The number of trees included for the proposed model implementation of random forest fuzzy entropy is 20. The accuracy obtained from the proposed Random Forest Fuzzy Entropy is 98 percent.

### 3.9. Results and performance evaluation

The experimental findings and performance evaluation of the suggested RFFE model are summarized in this section. The PID dataset is divided into 80:20 in this approach, with 80 percent of the data used to train the models and 20 percent used to verify their correctness. The performance is evaluated using precision, accuracy, recall/sensitivity, using precision, accuracy, recall/sensitivity, CNN-SGDM is used to evaluate the effectiveness of machine learning prediction systems. Oversampling of data has been carried out for the dataset with the use of SMOTE. This study adopts four classifiers, such as SVM, CART, KNN and NB, to compare with the proposed RFFE model for predicting diabetes diseases. This study evaluates the roots-mean-square-error (RMSE) to measure the prediction error rates.

The predictions' true/false positive/negative rate is investigated using the confusion matrix and ROCAUC curve. The PID dataset contains 768 patients' details, of which 20% data is taken for testing. The approximate test data is 154 patients' details, and it is presented in the confusion matrix with the distribution of patients having diseases, patients not having diseases, false prediction of patients not having diseases and false prediction of patients having diseases for SVM, CART, KNN, NB and RFFE algorithm. The detailed confusion matrix is depicted in Table 3.

**Table 3.**
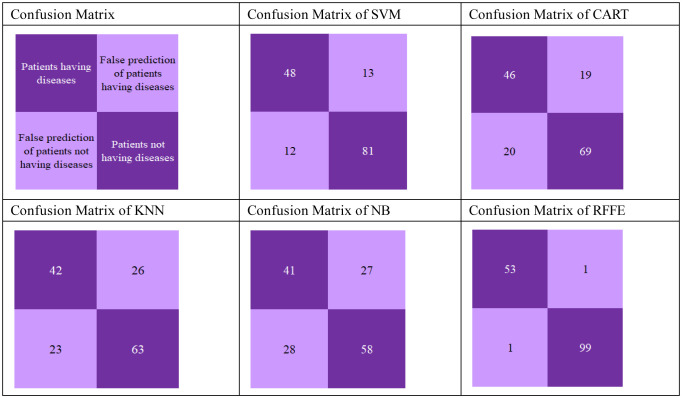
Detailed Confusion Matrix.

**Figure 5.**
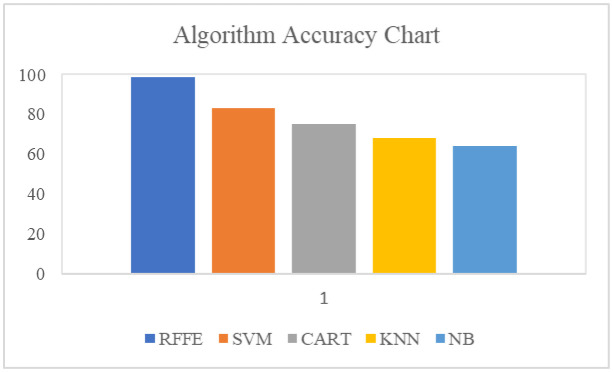
Accuracy.

The bar chart in Figure 5 compares the prediction accuracy algorithms. The accuracy for all the algorithms can be calculated by adding Patients having diseases and Patients not having diseases and dividing by the sum of patients having diseases and patients not having diseases, false prediction of patients not having diseases and false prediction of patients having diseases. The proposed RFFE model attained an accuracy of 98%, SVM achieved an accuracy of 84%, CART attained an accuracy of 75%, KNN attained an accuracy of 68% and NB attained an accuracy of 64%.

The comparison of performance metrics of accuracy, precision, recall and f1-score are given in Table 4.

**Table 4.** Performance metric.

S.No	Algorithm	Accuracy	Precision	Recall	f1-score
1	SVM	84	78.68	80	79.33
2	CART	75	70.76	69.69	70.23
3	KNN	68	61.76	64.62	63.16
4	NB	64	60.29	59.42	59.85
5	Proposed RFFE	98	98.14	98.13	98.12

**Figure 6.**
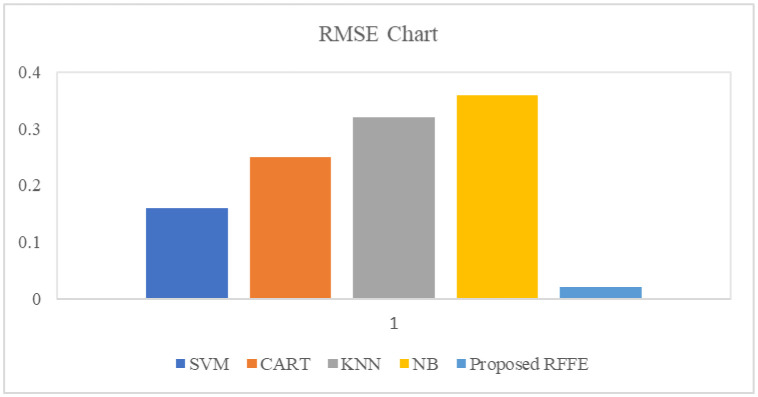
Error performance chart.

Error performance measures are also used to evaluate the erroneous in the algorithm. Figure 6 illustrates the error performance chart. It reveals that the suggested RFFE algorithm's root mean squared error (RMSE) is as low as 2%, whereas the RMSE of the SVM, CART, KNN and NB algorithms are 16 percent, 25 percent, 32 percent and 36 percent, respectively. As a result of the suggested feature selection approach, the proposed RFFE algorithm achieves a lower prediction error. It leads to the best prediction in accuracy.

**Figure 7.**
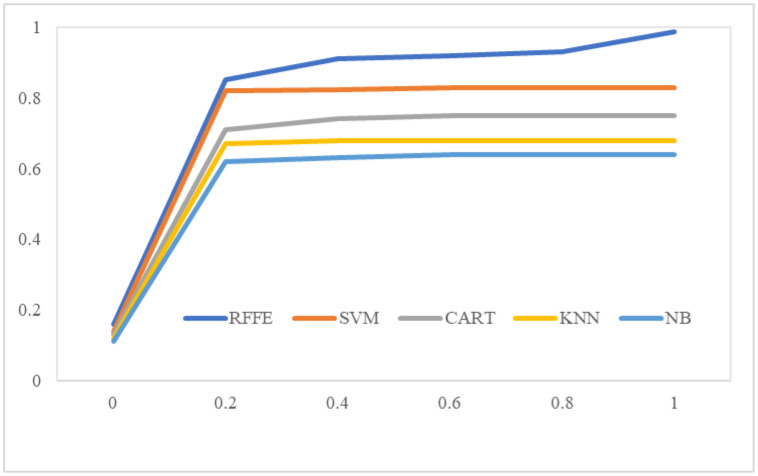
ROC of all models.

The ROC plot is a metric for assessing each algorithm's classification performance. Using ROC charts in medical diagnosis and prognosis has proven highly effective. A suitable test technique will have reference points in the ROC chart's top left corner. These points indicate that the reference values are very sensitive and have a low rate of false positives. Figure 7 shows that RFFE's AUC value of 0.98 is higher than the others.

**Figure 8.**
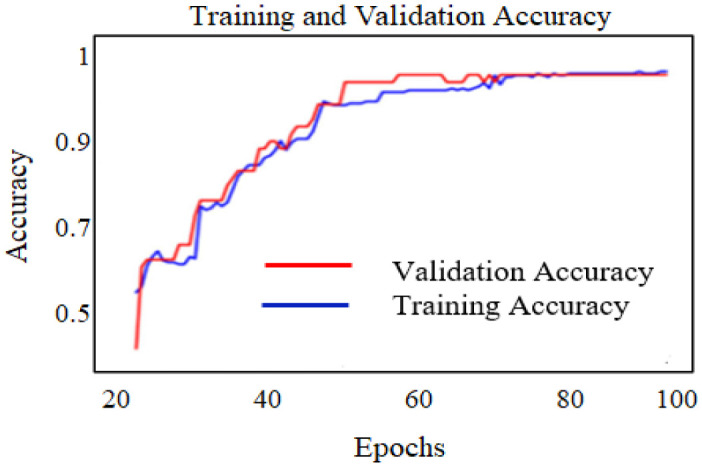
Accuracy performance of RFFE model.

**Figure 9.**
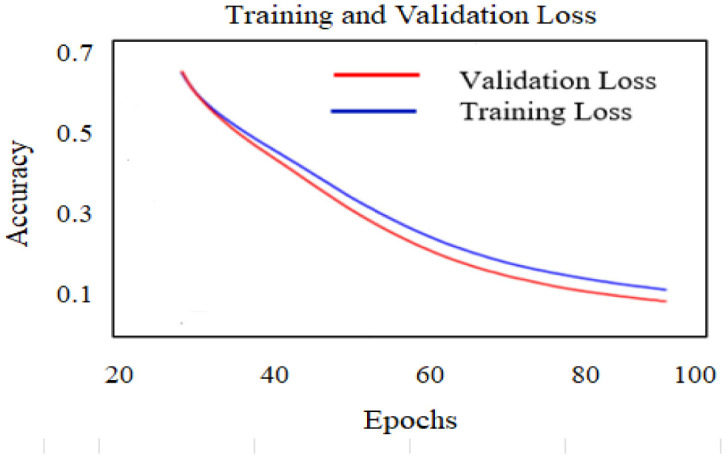
Loss performance of RFFE model.

The feature is trained on the RFFE algorithm with 100 epochs, the training and validation accuracy and loss are illustrated in Figures 8 and 9.

## 4. Conclusion

The primary research focuses on developing computational approaches and algorithms for illness diagnostics. A well-known diabetic disease dataset (PID) was used to predict the disease in this study. This research aims to increase the efficiency of feature pre-processing by employing a fuzzy entropy technique. As part of the DM diagnostic study, the random forest fuzzy entropy technique-based feature selection method is unveiled to increase the classification performance of a learning model. We compare our findings to well-known machine learning methods, including SVM, CART, KNN and NB. The RFFE model's computational findings indicate that fewer characteristics are required and can attain greater prediction accuracy of 98%. In future studies, an auto-tune machine learning programming architecture, which includes the number of hidden nodes and layers and the activation functions, to achieve higher performance; or optimize the parameters of the feature selection technique to get better performance can be incorporated.
